# AtlasT4SS: A curated database for type IV secretion systems

**DOI:** 10.1186/1471-2180-12-172

**Published:** 2012-08-09

**Authors:** Rangel C Souza, Guadalupe del Rosario Quispe Saji, Maiana OC Costa, Diogo S Netto, Nicholas CB Lima, Cecília C Klein, Ana Tereza R Vasconcelos, Marisa F Nicolás

**Affiliations:** 1 The National Laboratory for Scientific Computing LNCC, Brazil Av. Getúlio Vargas, Petrópolis, Rio de Janeiro, 333, Brazil

## Abstract

**Background:**

The type IV secretion system (T4SS) can be classified as a large family of macromolecule transporter systems, divided into three recognized sub-families, according to the well-known functions. The major sub-family is the conjugation system, which allows transfer of genetic material, such as a nucleoprotein, via cell contact among bacteria. Also, the conjugation system can transfer genetic material from bacteria to eukaryotic cells; such is the case with the T-DNA transfer of *Agrobacterium tumefaciens* to host plant cells. The system of effector protein transport constitutes the second sub-family, and the third one corresponds to the DNA uptake/release system. Genome analyses have revealed numerous T4SS in Bacteria and Archaea. The purpose of this work was to organize, classify, and integrate the T4SS data into a single database, called AtlasT4SS - the first public database devoted exclusively to this prokaryotic secretion system.

**Description:**

The AtlasT4SS is a manual curated database that describes a large number of proteins related to the type IV secretion system reported so far in Gram-negative and Gram-positive bacteria, as well as in Archaea. The database was created using the RDBMS MySQL and the Catalyst Framework based in the Perl programming language and using the Model-View-Controller (MVC) design pattern for Web. The current version holds a comprehensive collection of 1,617 T4SS proteins from 58 Bacteria (49 Gram-negative and 9 Gram-Positive), one Archaea and 11 plasmids. By applying the bi-directional best hit (BBH) relationship in pairwise genome comparison, it was possible to obtain a core set of 134 clusters of orthologous genes encoding T4SS proteins.

**Conclusions:**

In our database we present one way of classifying orthologous groups of T4SSs in a hierarchical classification scheme with three levels. The first level comprises four classes that are based on the organization of genetic determinants, shared homologies, and evolutionary relationships: (i) F-T4SS, (ii) P-T4SS, (iii) I-T4SS, and (iv) GI-T4SS. The second level designates a specific well-known protein families otherwise an uncharacterized protein family. Finally, in the third level, each protein of an ortholog cluster is classified according to its involvement in a specific cellular process. AtlasT4SS database is open access and is available at http://www.t4ss.lncc.br.

## Background

Knowledge about types of secretion pathways in prokaryotes has proportionally increased with the number of complete genomes deposited in the nucleotide databases. Moreover, several studies of secretion systems have been conducted with the purpose of understanding the biological mechanisms involved in the association between microorganisms and their hosts, since several secretion systems in prokaryotes should be mediating the mutualistic symbiotic or pathogenic relationships.

Secretion systems have been classified into seven major evolutionarily and functionally related groups, termed types I-VII [1-6]. Type IV Secretion System (T4SS) is one of the most functionally diverse, both in terms of the transported substrate (DNA, proteins, or DNA-protein complex) and the projected recipients (receiver cells or extracellular medium) [7]. According to this high range, three types of T4SS have been described: (i) the conjugation system (translocates DNA-protein substrates to recipient cells via a contact-dependent process) [8]; (ii) the effector translocator system (delivers proteins or other effector molecules to eukaryotic target cells) [9]; and (iii) the DNA release or uptake system (translocates DNA to or from the extracellular milieu) [10]. To accomplish that transport, the system comprises multisubunit cell-envelope-spanning structures, which form a secretion channel and often a pilus. Moreover, other proteins not needed for the assembly of the channel are required for the proper function of the system [11].

Most studies on T4SS have been carried out in some Gram-negative bacteria used as models: (i) the archetypal VirB/D4 encoded by pTi plasmid of *Agrobacterium tumefaciens*[12]; (ii) the *Helicobacter pylori* ComB that secretes DNA to the extracellular milieu [13]; (iii) Tra/Trb encoded by F plasmid of *Escherichia coli*[14]; and (iv) Dot/Icm identified in *Legionella* spp [15] and *Coxiella burnetti*[16] and (v) Tfc in genomic islands of *Haemophilus* spp [17]. Currently, there is information on a few T4SS subunits of Gram-positive bacteria, which are mainly representative of conjugation systems [18]. Also, a small number of archaeal conjugation systems have been recently described, such as the conjugative plasmids of thermophilic crenarchaeal *Sulfolobus* spp [19].

Nowadays it is generally accepted that the ancestral T4SS has evolved towards achieving a wide variety of biological activities, controlling genome architectures and interspecies relationships for novel purposes relating to the ongoing dialogue between donor and target cells [20]. The best model showing the sophisticated evolution and complexity of the T4SS is the VirD4/D4_pTi_ system, which has acquired many regulatory mechanisms to transport either virulence factors (VirE2, VirF), or a nucleoprotein complex (VirD2-T-DNA complex) to plant cells [21]. Another example is the *Legionella vir* homologue system (Lvh), which is partially required for conjugation and that can also act as an effector translocator involved in a virulence-related phenotype, under conditions mimicking the spread of Legionnaires' disease from environmental niches [22,23].

To date, the most accepted T4SS classification is based on the division of the systems into four groups [24]: (i) F-T4SS (Tra/Trb), (ii) P-T4SS (VirB/D4), (iii) I-T4SS (Dot/Icm), and (iv) GI-T4SS (T4SS that is found so far associated exclusively with genomic islands). This classification provides a framework for classifying most T4SSs. Despite this classification, unfortunately the proper genes nomenclature has not been standardized yet among the four groups. For example, there are several genes belonging to the F-T4SS group that are named *tra* or *trb* and the same nomenclature is used for some genes belonging to the P-T4SS group. Also, several orthologs of the Dot/Icm system identified in the Plasmid Collb-P9 have also been termed *tra* genes instead of dot/icm homologs. Alternatively, there are some examples showing that a particular T4SS group subunit has homology with a subunit of another T4SS group. That is the case of the DotB subunit of the I-T4SS group in *L. pneumophila,* which is homolog of P-T4SSs VirB11 [22]. Interestingly, deletion experiments in *L. pneumophila* show that the DotB protein can be replaced by the subunit LvhB11 to perform the conjugation process in this bacterium [22]. Hence, the ATPase DotB family [InterPro:IPR013363] shares the Type II secretion system protein E domain [Interpro:R001482), which is also found in the ATPase VirB11 family [Interpro: IPR014155]. Thus, it seems that DotB is a T4SS subunit more related to the P-type group than to the I-type group. Consequently, such cases make it difficult for researchers to decide, for instance, which one of the T4SS groups should be assigned for a given coding sequence (CDS) under a process of genome annotation.

In order to integrate the knowledge about Type IV Secretion Systems into a selected collection of curated data, we developed a comprehensive database that currently holds 134 ortholog clusters, totaling 1,617 predicted proteins, encoding the T4SS proteins organized in a hierarchical classification. This curated data collection is called AtlasT4SS - the first public database devoted exclusively to this type of prokaryotic secretion system.

## Construction and content

### Data sources and generation

The AtlasT4SS integrates into a centralized open access reference database of a collection of annotated coding sequences for prokaryotic T4SS proteins, organized in a hierarchical classification with three levels. Our dataset came from 58 Bacteria (49 Gram-negative and 9 Gram-Positive), one Archaea and 11 plasmids, downloaded from the NCBI ftp server [25]. Starting with these genome sequences, we looked for orthologous genes from a bi-directional best hit (BBH) relationship in a pairwise genome comparison [26]. Therefore, the orthologs were identified as BBH with BLASTP [27], in all-by-all comparisons of 70 genomic sequences. We extracted only target clusters, by using some keywords regarding the NCBI product or gene name related to T4SSs. Consequently, the final dataset contains 134 ortholog clusters totaling 1,617 predicted proteins encoding T4SS proteins.

### Database construction and annotation

**Figure 1:**
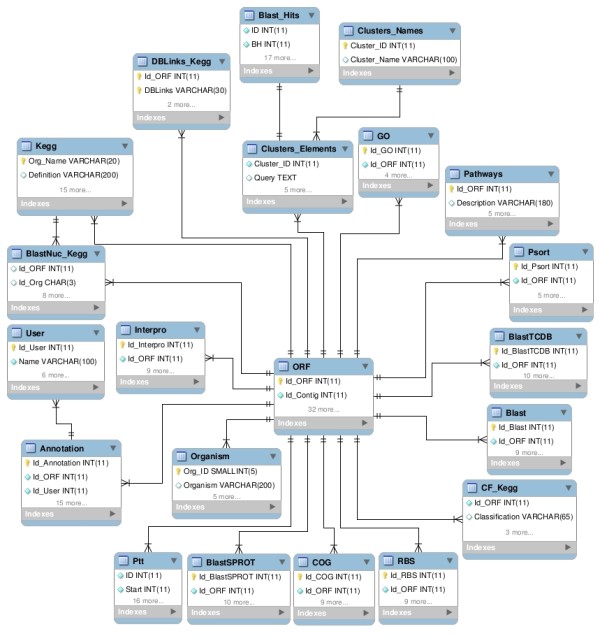
**Entity–relationship diagram of T4SS database.** Entities are represented by boxes and relationships by lines joining the boxes. The general information of the genes found in the ORF entity. Each entity ORF is related to information from biological database (InterPro, Swiss-Prot, Kegg, etc.) and tools (Psort, Phobius, etc.). Gene annotations and annotator entities are described in Annotation and User, respectively. The identified clusters are described by the entity Clusters_Names.

The AtlasT4SS database runs on a SUN-OS web server hosted by The National Laboratory for Scientific Computing (LNCC), Brazil. We used MySQL (v. 3.23.46) as a supported Relational Database Management System (RDBMS) to develop a database schema for storing sequence data, features, and annotation (Figure 1). The sequences, features and annotations are introduced into the database using Perl-based scripts with a web interface (HTML/CGI). Currently, the access to the database is done through the Web Perl-based Catalyst Framework.

**Figure 2:**
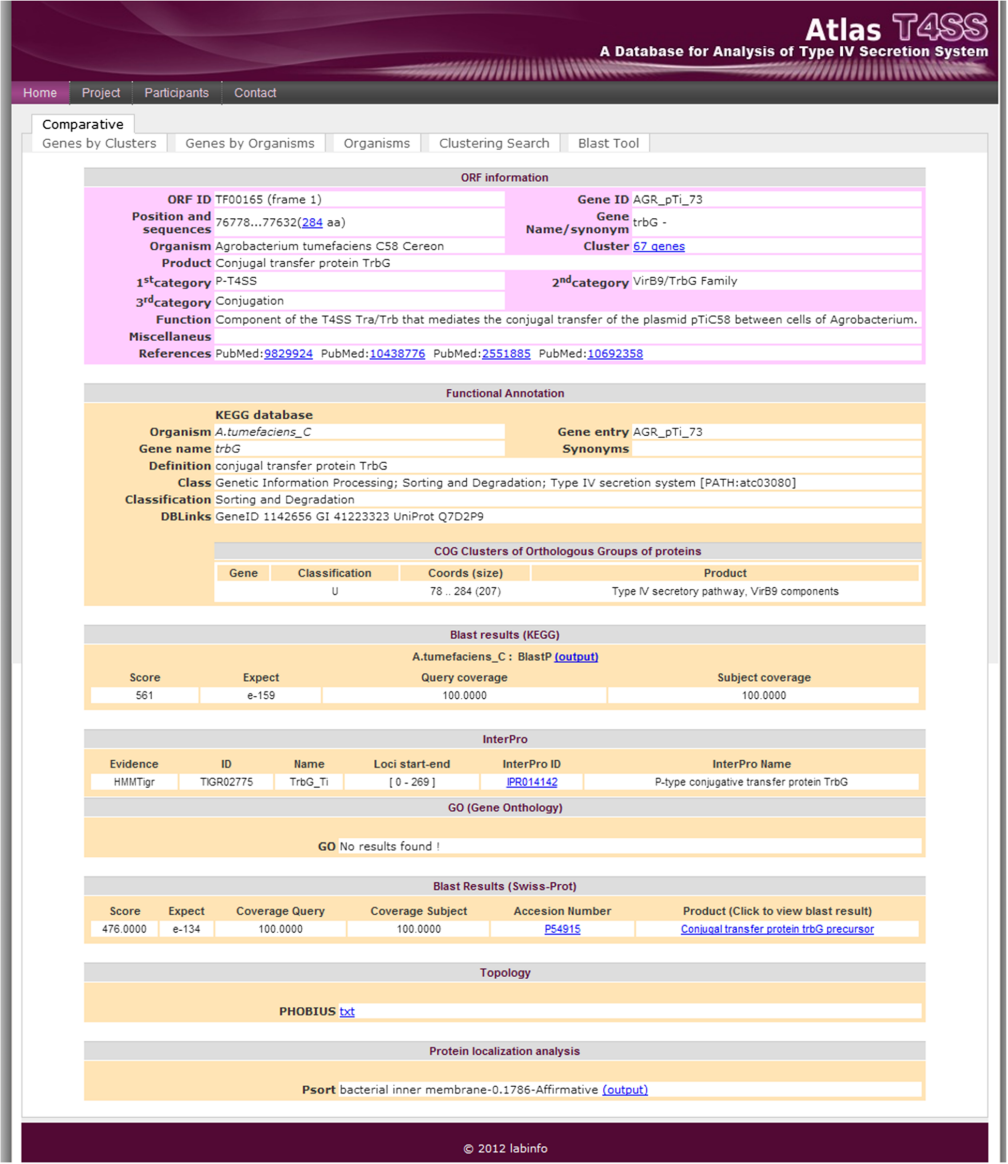
**Overview of annotation page of T4SS database.** The image provides an example of the main data page for a T4SS entry. Primary identification and annotation data appear at the top of the page with a link to the amino acid sequence as well as the corresponding T4SS cluster. This is followed by functional annotation data, which provide information by Kegg and COG, including the blast results against Kegg database. Below this are the sections containing details about the InterProScan, Gene Ontology (GO) as well as the blastp results against UniProt/Swiss-Prot database. Finally, the page shows details about the amino acid sequence topology and protein subcellular location prediction.

For annotation analysis, we applied the software SABIA (System for Automated Bacterial Integrated Annotation) [28] and ran several programs, including BLAST [27], CLUSTAL W Multiple Sequence Alignments package [29], MUSCLE (v. 3.6) [30] and Jalview (v. 2.3) [31]. Also, each T4SS record was submitted to several databases, such as InterPro [32] for protein domain and family annotation, KEGG (Kyoto Encyclopedia of Genes and Genomes) [33], COG (Clusters of Orthologous Groups of proteins) [34], gene onthology GO [35] and UniProtKB/Swiss-Prot [36] for functional classification, PSORT [37] for protein localization and Phobius [38] for protein topology features. Finally, we manually processed all automatic information obtained, including PubMed reference articles, in order to reach a final high quality annotation for each T4SS record (Figure 2).

## Utility and discussion

Our objective was to build an open access reference database to provide access to several proteins related to T4SS. To date, the AtlasT4SS holds 134 ortholog clusters. Their features are shown in Additional file 1: Table S1 that includes the presence of signal peptide and transmembrane regions, subcellular location and genomic location. These features were extracted from PubMed references, as indicated in the table, or from prediction algorithms.

### How to access the AtlasT4SS

**By “List of Biological sources”:** The list of biological sources contains 58 Bacteria (49 Gram-negative and 9 Gram-Positive), one Archaea and 11 plasmids, all known to carry at least one T4SS related protein. The list provides the TaxID NCBI number of each source and the link to the NCBI Taxonomy database.

**By “Genes by Clusters and Genes by Biological sources”:** The table of genes by clusters displays the 1^st^ T4SS category, the list of clusters, the biological sources compounding the cluster, the annotated product name, the gene ID - according to the NCBI- , and the CDS size. On the other way, the table of genes by biological sources gives almost similar information, sorting by biological sources instead of clusters.

We used controlled vocabulary in order to annotate the names of genes and products. For product name, we used two major denominations: (i) “Type IV secretion system protein”, for all proteins involved in effector translocation, T-DNA translocation or DNA Uptake/Release processes or, (ii) “Conjugal transfer protein”, for all proteins involved in the conjugation process. These denominations were according to the nomenclature used in the reference databases (UniProtKB/Swiss-Prot, COG, Kegg) or the cited literature. We added “homolog” as a final tag of the product name, to describe an ortholog system of one given archetypal T4SS system. For almost all gene names, we used the existing denomination found in NCBI or UniProtKB/Swiss-Prot.

**The “1st category":** We defined the first category according to the four well-known T4SS groups, as follows: (i) the **F-T4SS group** displays the Tra/Trb orthologs that form the conjugal transfer system encoded on the plasmid F identified in the *E. coli* genome; (ii) the **P-T4SS group** includes the Tra/Trb proteins that are encoded on the plasmids belonging to the incompatibility group IncP. This group also contains the orthologs of the archetypal *A. tumefaciens* VirB/D4 system, including the proteins Mpf (VirB subunits of the matting pair formation complex), T4CP (coupling-protein VirD4), and Dtr (Tra, VirC and VirD proteins that are involved in the DNA processing and its transfer to the Mpf/T4CP complex); (iii) the **I-T4SS group** includes ortholog clusters related to the archetypal *L. pneumophila*, *C. burnetti* and/or Plasmid Colb-P9 Dot/Icm systems; and (iv) the **GI-T4SS group** contains orthologs encoded on the genomic islands of *H. influenza*, *P. aeruginosa* and *Salmonella enterica*.

**The "2nd category":** The second category describes a well-known protein family or else an uncharacterized protein family (UPF). At present, the AtlasT4SS shows a total of 119 annotated protein families.

**The "3rd category":** The last category displays the classification based broadly on the function of a particular type IV secretion system. We described ten functional categories. When the function of a T4SS is well-known, we annotated it as either: (i) conjugation, (ii) effector translocator, (iii) T-DNA translocator, or (iv) DNA uptake/release. Also, when there is experimental evidence of bifunctional proteins, we annotated them with both functions, as follows: (v) conjugation and effector translocator or (vi) effector and T-DNA translocator. On the other hand, there are some uncharacterized systems, which we annotated as a probable function by analysis of similarity data (subject and query coverage ≥80% and similarity ≥80%) and phylogenetic tree, as follows: (vii) probable effector translocator, (viii) probable conjugation or (ix) probable effector translocator and DNA uptake/release. Finally, when the function of a given system was not possible to predict, we annotated it as (x) unknown.

**Figure 3:**
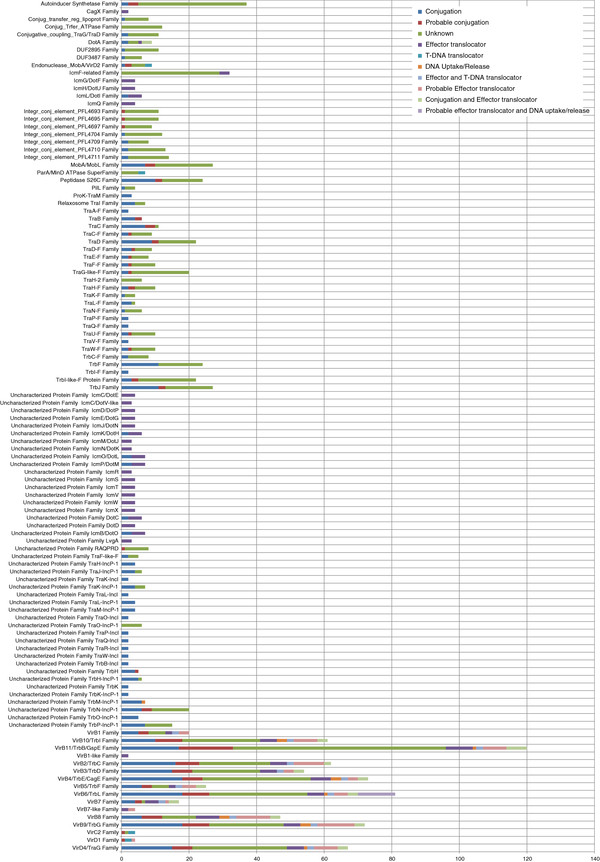
**Distribution of family sizes in the Atlas T4SS.** The graphic shows the distribution of the 119 protein families annotated in the 2^nd^ category of the Atlas T4SS according to the number of entries per family. The colors within each bar indicate the percentage of entries annotated with a known or unknown function.

The current version of the AtlasT4SS database contains 119 families dispersed into 134 clusters. Each protein family can be related to one cluster (*e.g.* F-T4SS TraA-F family), two clusters (*e.g.* I-T4SS DotA family), three clusters (*e.g.* P-T4SS VirB7 family), or up to eight clusters (*e.g.* P-T4SS VirB2/TrbC family). Figure 3 shows the distribution of protein family sizes in the database, and for each of them its functional category is highlighted. This figure allows a simple identification of functional category within a given family. For example, the largest protein families (more than 10 members), in particular those belonging to the P-T4SS group contain several annotated functional categories, including the unknown function. These functional categories vary from four for Endonuclease_MobA/VirD2 Family to eight for several VirB related families and nine for VirB6/TrbL Family.

### Clustering search mode

**Figure 4:**
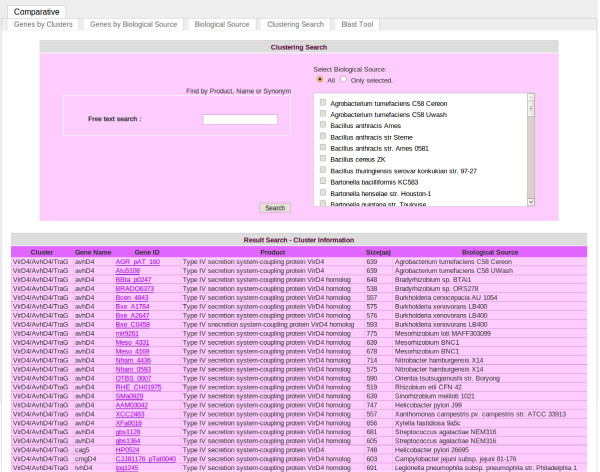
**Clustering search tool of T4SS database.** The image provides an example of the clustering search tool results with the keyword “virD4” in *Agrobacterium tumefasciens* C58 Cereon.

**Figure 5:**
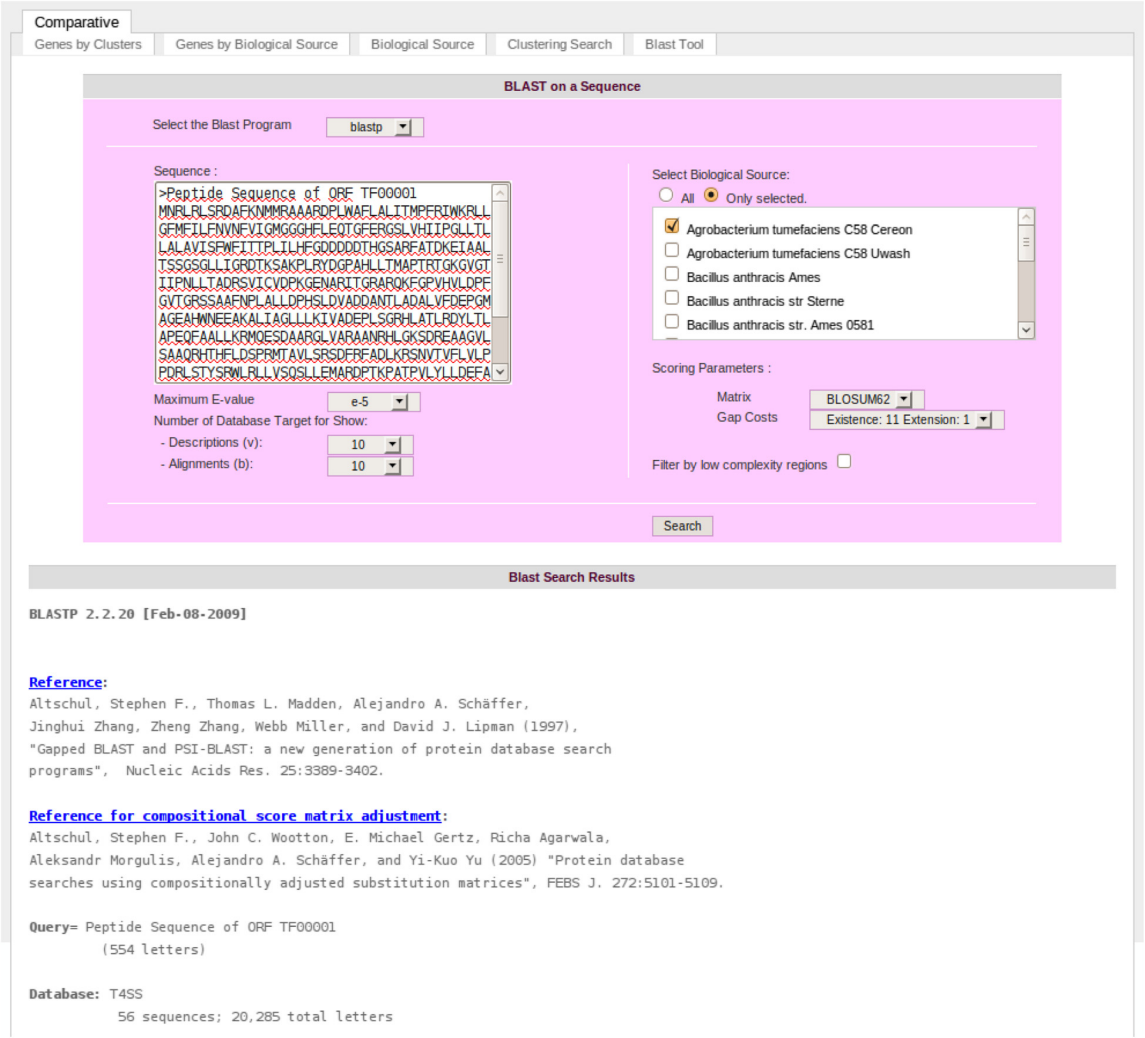
**Blastp tool of T4SS database.** The image provides an example of the blastp results with an unknown amino acid sequence query against the complete genome sequence of *Agrobacterium tumefasciens* C58 Cereon.

**Figure 6:**
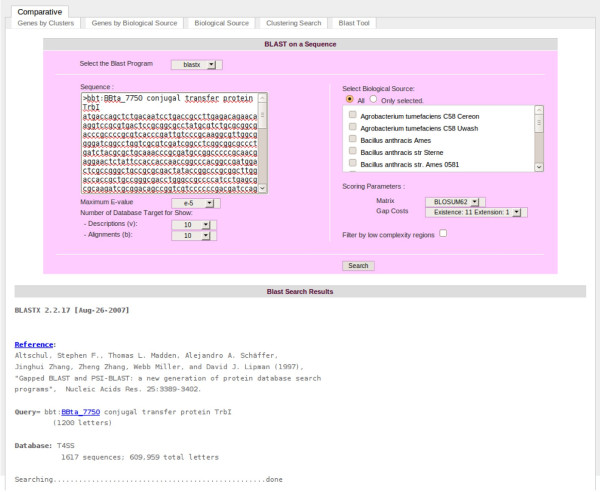
**Blastx tool of T4SS database.** The image provides an example of the blastx results with an unknown nucleotide sequence query against all biological sources of Atlas T4SS.

This mode corresponds to an advanced search with several parameters that allow the user to retrieve selected T4SS data using one or more filtering parameters. Moreover, this searching tool is a comparative mode, since the user can select biological sources of interest from the whole list. Thus, the user can retrieve T4SS records by entering the product, gene name or synonym (by NCBI gene ID). Also, it allows performing a search by either selecting an interesting biological source(s) or from the whole list of biological sources. Figure 4 shows an example of a search: T4SS proteins involved in conjugation belonging to the VirD4/TraG family in *A. tumefasciens* C58 Cereon, *Rhizobium etli* CFN 42 and *Mesorhizobium loti* R7A. It is also possible to run a BLASTP and BLASTX algorithm with a query amino acid or nucleotide sequence against AtlasT4SS clusters (Figures 5 and 6).

### Phylogenetic analysis

Using the concatenated amino acid sequences of the ortholog clusters containing three or more predicted proteins, we generated a NJ midpoint-rooted trees for each ortholog cluster. A total of 108 phylogenetic trees are displayed in the AtlasT4SS. Overall, all clusters represent a mixture of described functions, including effector translocators, DNA uptake/release and conjugation systems. However, a closer examination of the major trees resulting from alignment of amino acid sequences encoded by VirB1/AvhB1, VirB2/AvhB2, VirB3/AvhB3, VirB4/AvhB4/TrbE/CagE, VirB6/AvhB6/TrbL, VirB8/AvhB8, VirB9/AvhB9/TrbG, AvhB10/VirB10/TrbI, AvhB11/VirB11/TrbB/GspE, VirD4/AvhD4/TraG and their homologues revealed that single branches grouped proteins with the same functional classification.

Accordingly, these T4SS trees display two categories of functions: single branches grouping effector translocator systems, and the other ones grouping conjugation systems. For example, the midpoint-rooted phylogenetic tree of the AvhB11/VirB11/TrbB/GspE cluster [39] contains the highest number of sequences, totalizing 206, including 142 paralogs. As mentioned before, proteins VirB11 belong to the ATPase VirB11 family, which contains the Type II secretion system protein E domain, also found in the DotB family. Consequently, the BBH merged into the same cluster, VirB11, TrbB, and also the GspE proteins of type II (e.g., GeneID: lpg1522 and product: Type IV fimbrial assembly protein *pil*B), but these sequences were not included in this tree. It is important to note that the VirB11 homolog from *Campylobacter jejuni* (CJJ81176pTet0039) involved in DNA uptake/release is closer to the conjugative TrbB proteins, which is also observed in the VirB4 phylogenetic tree [40].

There is only one discrepancy in the grouping of functions at the final branches: the VirB11 from *Brucella suis* (BRA0059), which is an effector translocator system, was grouped on the same branch of TraM protein from a possible conjugative plasmid pSB102. Hence, this discrepancy is observed in all phylogenetic trees of the P-T4SS clusters.

### A case study: T4SS in *Rhizobium etli* CFN42

The genome of *R. ettli* strain CFN42, a nitrogen-fixing bacterium, consists of one chromosome and six plasmids, and contains three copies of the T4SS: the plasmid p42a carries two copies of T4SSs (VirB/D4p42a and Tra/Trbp42a), and the symbiotic plasmid p42d carries one VirB/D4p42d system [41].

The Tra/Trbp42a is involved in conjugal transfer of the self-transmissible plasmid p42a, and can mobilize the symbiotic plasmid p42d. On the other hand, the VirB/D4p42d probably is not a functional conjugation system [41]. Concerning the function of the third T4SS, the VirB/D4p42a, we postulated the hypothesis that this system is a possible effector translocator. Through examination of the phylogeny of ortholog clusters, we observed that all VirB/D4p42a subunits grouped together with the effector translocator systems VirB/D4Ti of *A. tumefasciens* and VirB/D4pR7 of *Mesorhizobium loti*. The alphaproteobacteria *M. loti* belonging to the Rhizobiales order enables symbiotic relationships for biological nitrogen fixation with *Lotus* spp., including *Lotus corniculatus* and the model legume plant *L. japonicus*. The *M. loti* VirB/D4pR7 is encoded in the symbiotic island of plasmid R7A, and was proven to be an effector translocator system, essential for plant symbiosis [42,43]. To date, two substrates transferring by the VirB/D4pR7 to the host plant have been identified *in vitro*, one being the product of ORF msi059, and the other one the product of ORF msi061 [42]. This T4SS is the first example of a type IV being involved in mutualistic symbiotic relationships.

Interestingly, looking for msi059 and msi061 homologues in the *R. etti* CFN42 genome, we found two ORFs in the plasmid p42a. One is RHE_PA00030 (270 aa) belonging to the Peptidase C48 family, which is similar to a domain of msi059 (61% BLASTP over 15% of the length of the protein). The other one is RHE_PA00040 (203 aa) (annotated as VirF1), which is similar to msi061 (54% BLASTP over 42% of the length of the protein) and VirF (52% BLASTP over 78% of the length of the protein), a protein transferred by the VirB/D4_Ti_ required for *A. tumefasciens* virulence [44].

Consequently, according to evidence shown in our analysis, we suggest experimental investigation of VirB/D4p42a in order to elucidate the probable effector translocator function and its involvement in the *R. etti* CFN42 symbiosis. Through T4SS analysis of symbiotic bacteria, it is possible to verify a role of this system for the host relationship. Perhaps in these bacteria, the T4SS can replace the same secretion function mediated by another system, such as the type III secretion system.

### Future development and perspectives

Currently, we are working to include new systems and the related substrates for the effector translocator systems in the database. Also, we will perform an upgrade of the database to incorporate more systems from Gram-negative and Gram-positive Bacteria and Archaea.

## Conclusion

In summary, AtlasT4SS is a comprehensive and web-accessible database of type IV secretion system in prokaryotes. This is a public resource devoted to the knowledge about classification, function and evolution of this transport system from a variety of bacterial and archaeal genomes. AtlasT4SS will be useful for the annotation of T4SS in prokaryotic genomes.

## Availability and requirements

**Database name:** AtlasT4SS.

**Project home page:**http://www.t4ss.lncc.br.

**Operating system(s):** Platform independent.

**Programming languages:** AtlasT4SS is an interactive web-based database with user-friendly interface (HTML/Web-Based MVC). Information is provided using the RDBMS MySQL and the Catalyst Framework based in Perl programming language and Model-View-Controller (MVC) design pattern for Web Use by non-academics: no license needed.

## Abbreviations

T4SS: Type IV Secretion System; BBH: Bi-directional Best Hit; CDS: Coding Sequence; UPF: Uncharacterized Protein Family; Mpf: Matting pair formation complex; T4CP: Coupling-Protein VirD4; Dtr: DNA processing and transfer.

## Authors’ contributions

RCS, GRQS, DSN and MFN retrieved, analyzed, prepared the AtlasT4SS dataset (sequence, functional annotation, cross-references…) and illustrated the relational database. RCS and GRQS performed scripts for automated data retrieval and developed the current web pages. MFN, MOCC and CCK in cooperation carried out the CDS annotation and designed the T4SS hierarchical classification. NCBL worked on the phylogenetic trees figures. MFN and ATRV managed the project. ATRV is the team leader and provides financial support. All authors read and approved the final manuscript.

## Supplementary Material

Additional file 1 Table S1. Cluster's statistics information. Click here for file
